# Are Australian Mental Health Services Ready for Therapeutic Virtual Reality? An Investigation of Knowledge, Attitudes, Implementation Barriers and Enablers

**DOI:** 10.3389/fpsyt.2022.792663

**Published:** 2022-02-04

**Authors:** Olivia S. Chung, Alisha M. Johnson, Nathan L. Dowling, Tracy Robinson, Chee H. Ng, Murat Yücel, Rebecca A. Segrave

**Affiliations:** ^1^BrainPark, Turner Institute for Brain and Mental Health and Monash Biomedical Imaging Facility, Monash University, Melbourne, VIC, Australia; ^2^Professorial Unit, Department of Psychiatry, The Melbourne Clinic, The University of Melbourne, Melbourne, VIC, Australia; ^3^School of Nursing, Paramedicine and Healthcare Sciences, Charles Sturt University, Bathurst, NSW, Australia

**Keywords:** virtual reality, implementation, acceptability, appropriateness, feasibility, mental health

## Abstract

Therapeutic virtual reality (VR) has the potential to address the challenges of equitable delivery of evidence-based psychological treatment. However, little is known about therapeutic VR regarding the perspectives and needs of real-world service providers. This exploratory study aimed to assess the acceptability, appropriateness, and feasibility of therapeutic VR among clinicians, managers, and service staff working in mental healthcare and explore potential implementation barriers and enablers. Eighty-one staff from a network of private psychiatric hospitals in Victoria, Australia (aged *M* + *SD*: 41.88 + 12.01 years, 71.6% female; 64% clinical staff) completed an online survey, which included the Acceptability of Intervention Measure (AIM), Appropriateness of Intervention Measure (IAM), and Feasibility of Intervention Measure (FIM). While 91% of participants had heard about VR technology, only 40% of participants had heard of therapeutic VR being used in mental healthcare, and none had used therapeutic VR in a clinical setting. Most participants perceived VR to be acceptable (84%), appropriate (69%), and feasible (59%) to implement within their role or service and envisioned a range of possible applications. However, participants expressed concerns regarding safety, efficacy, and logistical challenges across clinical settings. Findings suggest a strong interest for therapeutic VR among Australian mental health providers working in the private system. However, dissemination efforts should focus on addressing identified barriers to ensure mental health providers are adequately informed and empowered to make implementation decisions.

## Introduction

With the burden of mental illness rising across all countries at a projected cost of $16 trillion to the global economy by 2030 (1), there is an urgent need for innovation to ensure more effective, equitable and timely access to mental health services. Leveraging digital technologies has been highlighted as a key strategy to meet unmet mental health needs (2, 3). However, implementation of digital health technologies into clinical practice has been challenging (4). Notably, the COVID-19 pandemic has catalyzed rapid adoption of digital and online platforms across healthcare systems that have previously been underutilized despite wide availability and demonstrated efficacy (5). For instance, in the US, over two-thirds of psychologists had never used videoconferencing to deliver services prior to 2018 (5). Within this new era of digital healthcare is an opportunity for therapeutic virtual reality (VR) to be integrated as an innovative treatment tool.

VR is an immersive technology that uses computer-generated, 3D-environments to transport people into realistic scenarios, typically through a head-mounted display (HMD) (6). By effectively engaging participants' natural sensorimotor contingencies, VR creates a sense of presence (i.e., illusion of “being there”) and embodiment (i.e., illusion of virtual body ownership) (7, 8), which can be exploited to enhance traditional psychological therapies. Research over two decades has investigated VR's potential to provide systematically controllable, ecologically valid environments to safely modify maladaptive cognitive, emotional, and physiological responses (9). Presently, over 30 randomized control trials support VR exposure therapy (VRET) as an effective treatment for anxiety-related disorders (i.e., phobias, post-traumatic stress disorder (PTSD), panic disorder), with equivalent effect sizes (10) and attrition rates compared to *in vivo* exposure therapy (11, 12). Moreover, there is growing evidence supporting its utility in assessing and treating a broader range of conditions including autism-spectrum disorders, addiction, depression, eating disorders, pain, and psychosis (13, 14). The potential benefits of VR are numerous. For instance, VR enables patients greater opportunity for direct learning with the support of a clinician in simulations of real world situations where their psychological difficulties occur may accelerate treatment gains (15). VR can also address practical challenges associated with accessing relevant therapeutic stimuli (e.g., phobic stimuli), which can be impractical, costly, or even risky to recreate in conventional clinical settings (16). VR may also be a more acceptable treatment approach (e.g., compared to *in vivo* exposure) to patients (17), which may encourage earlier or sustained help-seeking in patients who find traditional treatment aversive.

While technology barriers (e.g., high cost, heavy apparatus, unreliable performance) have previously hindered meaningful translation from research to clinical settings, the release of consumer HMDs in 2016 has greatly altered implementation considerations. Since then, the technology has rapidly evolved, with entry-level headsets providing more immersive, ergonomic VR experiences (i.e., enabled by six degrees of freedom head-tracking, refresh rates of ≥90 Hz, wider field of view) than their predecessors at increasingly accessible price-points (Table 1 provides an overview of most popular VR systems commercially available and newly released). Concurrently, the therapeutic VR market has expanded, with several vendors offering various subscription plans (see Table 2). Yet, despite demonstrated efficacy and improved technology availability, therapeutic VR has seen limited uptake in mainstream clinical settings (28, 29). This reflects the broader pattern of slow evidence-based practice (EBP) adoption by healthcare systems (30), as it is estimated that only about half of EBPs are successfully incorporated into routine clinical practice, taking 17 years on average (31). This “time lag” highlights the inherent complexities of practice and policy in local contexts. As the application of VR in mental healthcare is emerging, it is timely to better understand the perspectives of frontline stakeholders.

**Table 1:** Integrated immersive VR systems commercially available.

**Manufacturer and** **system**	**Release** **year**	**Standalone/** **tethered^a^**	**Head-tracking** **DoF**	**Horizontal** **FoV**	**Display (resolution per eye)**	**Refresh rate**	**Weight**	**Cost^b^**
HP Reverb G2	2021	Tethered	6DoF	114°	LCD (2,160 × 2,160)	90 Hz	550 g	$599
HP Reverb G1	2019	Tethered	6DoF	114°	LCD (2,160 × 2,160)	90 Hz	498 g	$599
HTC Vive Flow	2021	Standalone	6DoF	101°	LCD (1,600 × 1,600)	75 Hz	239 g	$499 (headset only)
HTC Vive Pro	2016	Tethered	6DoF	110°	AMOLED (1,440 × 1,600)	90 Hz	550 g	$1,199
HTC Vive Pro 2	2021	Tethered	6DoF	120°	LCD (2,248 × 2,248)	120 Hz	850 g	$1,399
HTC Vive Cosmos	2019	Tethered	6DoF	110°	LCD (1,440 × 1,700)	90 Hz	702 g	$699
HTC Vive Focus 3	2021	Standalone	6DoF	120°	LCD (2,448 × 2,448)	90 Hz	785 g	$1,300
Pico G2 4K	2019	Standalone	6DoF	101°	LCD (1,920 × 2,160)	75 Hz	470 g	$300
Pico Neo 3 Pro	2021	Standalone	6DoF	98°	LCD (1,832 × 1,920)	90 Hz	620 g	$699
Pico Neo 3 Pro Eye	2021	Standalone	6DoF	98°	LCD (1,832 × 1,920)	90 Hz	620 g	$899
Oculus Quest 2	2020	Standalone	6DoF	89°	LCD (1,832 × 1,920)	120 Hz	508 g	$299
Oculus Quest	2019	Standalone	6DoF	94°	OLED (1,440 × 1,600)	72 Hz	571 g	$399
Oculus Rift S	2019	Tethered	6Dof	90°	LCD (1,280 × 1,440)	80 Hz	561 g	$399
Pimax Vision 8K X	2019	Tethered	6DoF	150°	CLPL (3,840 × 2,160)	90 Hz	984 g	$1,599
Pimax Vision 5K Super	2020	Tethered	6DoF	150°	CLPL (2,560 × 1,440)	180 Hz	750 g	$1,299
Valve Index HMD	2019	Tethered	6DoF	130°	LCD (1,440 × 1,600)	144 Hz	809 g	$999
Varjo Aero	2021	Tethered	6DoF	115°	LCD (2,880 × 2,720)	90 Hz	710 g	$1,990 (headset only)
Varjo VR-3	2021	Tethered	6DoF	115°	uOLED (1,920 × 1,920), LCD (2,880 × 2,720)	90 Hz	558 g	$3,395 (headset only)
Varjo XR-3	2021	Tethered	6DoF	115°	uOLED (1,920 × 1,920), LCD (2,880 × 2,720)	90 Hz	980 g	$5,995 (headset only)

*Overview of integrated, fully immersive VR systems. The list is based on the most popular headsets by SteamVR usage (https://store.steampowered.com/hwsurvey/) as a proxy of the most stable and core products since the release of consumer VR headsets, and new system releases as of November 2021. DoF, degrees of freedom; FoV, field of view.*

a *Standalone HMDs are all-in-one devices with all necessary components to deliver VR experiences. Tethered HMDs serve as the display for another device (e.g., PC), which may be used cabled or wirelessly with an adapter.*

b *Starting prices in USD at time of search for base models with peripheral devices (e.g., hand controllers, base stations) unless otherwise specified*.

**Table 2:** Therapeutic VR software vendors for mental health.

**Company**	**Applications^a^**	**Evidence^b^**	**Cost**
AppliedVR	Pain	(18, 19)	N/A^d^
BehaVR	Stress reduction, pain, postpartum mood, and anxiety disorders (PMAD)	N/A	$599 USD for 22 weeks (PMAD)^c^
C2 Care	Addictions, GAD, eating disorders, OCD, PTSD, pathological gambling, phobias, social anxiety	N/A	$165 USD/month^b^
CleVR	Psychotic disorders, social anxiety	(20, 21)	N/A^d^
Cynergi Health	Addictions	N/A	N/A^d^
Mindcotine	Addictions	(22)	N/A^d^
Oxford VR	Psychotic disorders, Acrophobia	(15)	Not commercially available
Psious	Addictions, GAD, OCD, pain, phobias, relaxation, social anxiety	N/A	$165 USD/month^c^
Virtually Better	Addictions, phobias, PTSD (combat, military sexual trauma)	(23–25)	N/A^d^
Virtue Health	Dementia	N/A	N/A^d^
VRelax	Relaxation	(26)	$1,141^c^
XRHealth	Attention deficit hyperactivity disorder, cognitive training, pain, stress, relaxation	N/A	$69 USD/week^c^
ZeroPhobia	Acrophobia	(27)	Free software license only

*This list is not intended to be exhaustive, but rather to provide an overview of key companies in the industry, as of November 30, 2021.*

a *GAD, generalized anxiety disorder; OCD, obsessive-compulsive disorder; PTSD, post-traumatic stress disorder.*

b *While applications have been developed with evidence-based principles, few have completed testing in randomized controlled trials to demonstrate efficacy.*

c *Approximate starting price of subscription plan for one user (including hardware and software license). Custom subscription plans for enterprises (multiple account users) are available.*

d *Company to be contacted for program pricing*.

According to Proctor et al. (32), the acceptability, appropriateness, and feasibility of a novel EBP are the outcomes most valuable to assess during early-stage implementation, as they are essential pre-conditions for successful and sustained uptake. Acceptability is the perception that an EBP is agreeable or satisfactory (e.g., in content, comfort, complexity, credibility); appropriateness is the perception that the EBP is useful or compatible with a given setting, provider or consumer (e.g., clinical suitability, organizational mission); and feasibility is the degree to which an EBP can be successfully carried out within a given setting. To date, studies investigating therapeutic VR implementation are limited, with most conducted prior to 2016 and focused specifically on its application in exposure therapy, stroke rehabilitation or pain (33–39). Only one study has examined contemporary attitudes toward VRET among clinicians practicing cognitive behavioral therapy (CBT), with results indicating that concerns may have shifted from predominantly technology-related reservations (e.g., operating difficulties, poor immersion) to therapeutic efficacy (28). However, the perspectives of cross-disciplinary clinicians, service directors, managers, and administrators working in mental healthcare remain poorly understood, despite their strong influence on therapeutic VR integration into practice and management of related service operations. Focusing on providers working in the privately funded system is a strategic starting point as they are frequently earlier adopters of new evidence-based practices compared with public system settings (40).

The current study sought to survey knowledge, attitudes, and perceived implementation barriers and enablers for therapeutic VR among clinicians and non-clinical service staff working across a network of private psychiatry hospitals in Victoria, Australia. Two explorative research questions were investigated: (i) do clinicians and non-clinical staff find therapeutic VR to be acceptable, appropriate, and feasible to implement within mental healthcare? and (ii) what are the potential barriers to, and enablers for, the implementation of therapeutic VR in mental healthcare?

## Methods

### Ethics Statement

The study was approved by the Melbourne Clinic Human Research Ethics Committee (#304) and the Monash University Human Research Ethics Committee (#13284). Informed consent was implied through survey completion. The ethics and consent statement and survey questions are included in the Supplementary Material.

### Participants and Procedure

Eighty-one clinical and non-clinical health service staff (aged *M* = 41.88 ± 12.01 years; 71.6% female; 64% working in a clinical role) were recruited from the Healthscope network of private psychiatric hospitals in Melbourne, Victoria, as part of Australia's largest private healthcare provider for mental health issues and for substance use disorders. Participants were recruited from hospitals via site-specific staff email lists between May to September 2019, with the inclusion criteria being a current Healthscope staff member or student, no exclusion criteria were applied. The email included a brief explanation about the study and link to the online survey. At the end of the survey, participants were invited to enter their details for a prize draw of a 1x $200 gift-card in appreciation for their time and effort. Respondents worked across three hospital sites in metropolitan and regional Victoria, including The Melbourne Clinic (*n* = 63, 78%), The Geelong Clinic (*n* = 10, 12%), and The Victoria Clinic (*n* = 8, 10%). Table 3 presents additional participant characteristics.

**Table 3:** Participant demographics.

**Characteristics**	***N*** **(%)**	
	**Clinical** **(*n* = 52)**	**Non-clinical** **(*n* = 29)**
Role background		
Psychiatrist	7 (14)	
Psychologist	9 (17)	
Nurse	25 (48)	
Other allied health^a^	11 (21)	
Management		9 (35)
Administration		14 (48)
Research/student^b^		6 (21)
Years in role or similar prior role		
≤ 1y	6 (12)	6 (20.7)
2–5y	22 (42)	11 (37.9)
6–10y	11 (21)	7 (24.1)
11–15y	6 (12)	3 (10.3)
16–20y	3 (6)	0 (0.0)
>20y	4 (8)	2 (6.9)
Clinical setting worked in (multiple answers)		
Inpatient	31 (59.6)	
Outpatient	24 (38.5)	
Patient age groups worked with (multiple answers)^c^		
Youth (16–24 years)	21 (40.4)	
Adults (25–65 years)	42 (80.8)	
Older adults (≥65 years)	10 (19.2)	
Primary disorders worked with (multiple answers)^d^		
Addictions	9 (17.3)	
Anxiety disorders	34 (65.4)	
Bipolar and related disorders	7 (13.5)	
Depressive disorders	36 (69.2)	
Eating disorders	4 (7.7)	
Obsessive-compulsive related disorders	2 (3.8)	
Personality disorders	32 (61.5)	
Trauma-related disorders	13 (25.0)	
Psychotic disorders	1 (1.9)	
Age (years)		
20–29y	10 (19.2)	6 (20.7)
30–39y	15 (28.8)	5 (17.2)
40–49y	12 (23.1)	8 (27.6)
50–59y	10 (19.2)	7 (27.6)
≥60y	4 (7.7)	2 (6.9)
Not specified	1 (1.9)	0 (0.0)
Gender		
Male	17 (33)	6 (21)
Female	34 (65)	23 (79)
Prefer not to say	1 (.02)	0 (0)

a *Art therapist, counselor, dietician, physiologist, occupational therapist, social worker.*

b *Clinical researchers and medical/allied health students.*

c *Worked with ≥ 20% of typical week.*

d *Up to three disorders chosen*.

### Survey

The survey was delivered online in English *via* Qualtrics, median completion time was 17.1 min, and was subdivided into six sections (i.e., five response sections and one information section). The sections are described below in order of completion.

*I. Demographics*. This section included questions regarding participant demographics (e.g., age, gender) and their professional background (e.g., primary role, years in current or similar role, clinical settings worked in, and patient age groups and primary clinical diagnoses worked with).

*II. Prior knowledge and impression of VR*. This section included questions about participants baseline knowledge of VR, including whether they had heard of VR (“Yes,” “No”), previously tried VR (“Yes,” “No,” “Not sure”), where prior VR experiences occurred (e.g., personal home, gaming outlet, museum), and what their overall impression of VR was generally (“positive,” “neutral,” “negative”), prior to completing the survey. An optional short answer question was included to further explore participants' impressions.

*III. Prior knowledge and impression of therapeutic VR*. This section asked participants whether they had heard of therapeutic VR being used therapeutically in medicine, psychology, or psychiatry ("Yes,” “No”), used therapeutic VR with patients (“Yes,” “No”), and what their overall impression of VR as a therapeutic tool in mental healthcare was (“positive,” “neutral,” “negative”) prior to completing the survey. An optional short answer question was included to further explore participants' impressions.

**Figure 1:**
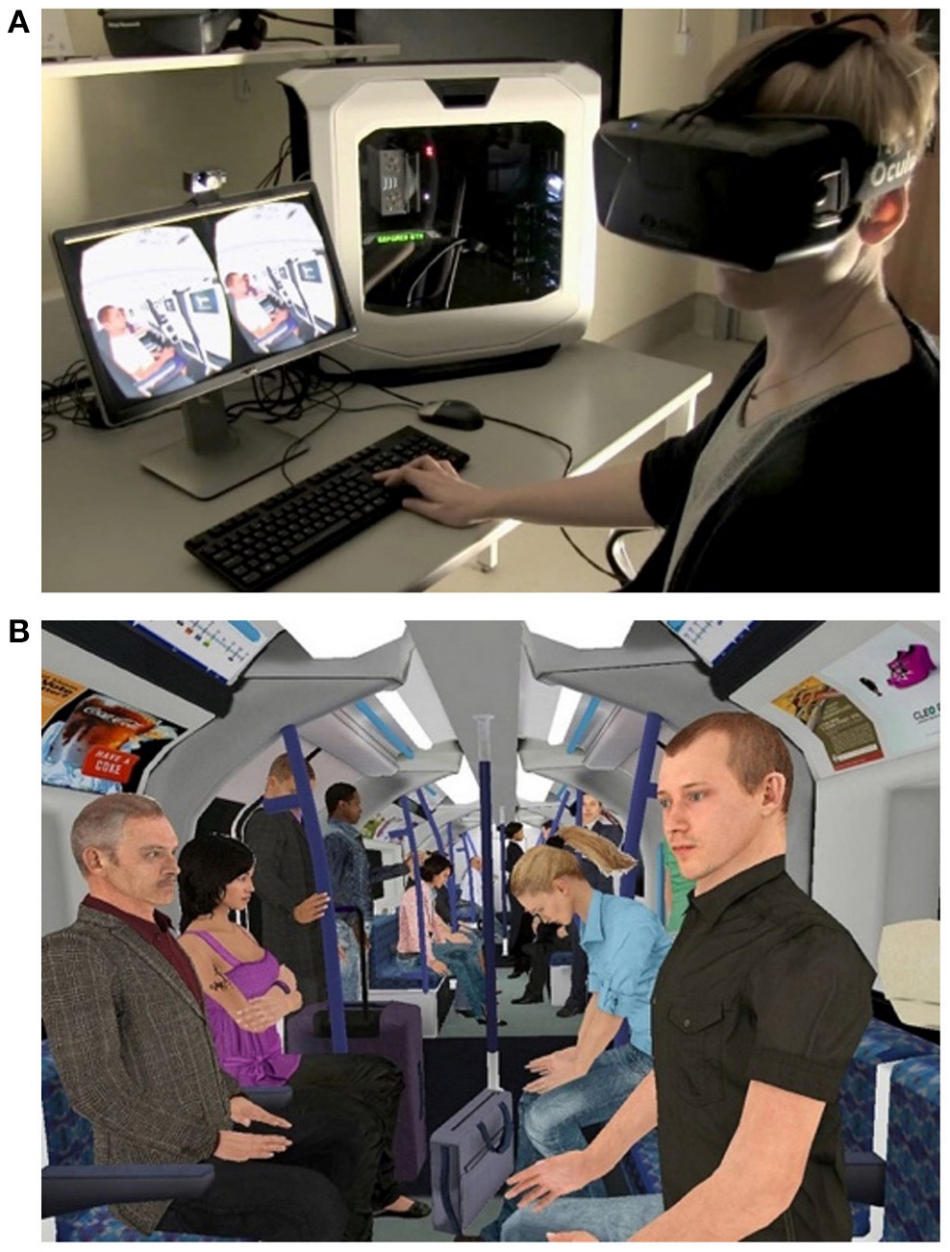
**(A,B)** Screenshots from the video shown to participants, demonstrating a therapeutic VR program developed for the treatment of persecutory delusions (University of Oxford).

*IV. Information section*. As VR and its applications in mental healthcare are still emerging, it was anticipated that participants' knowledge would be variable. Thus, to increase participants' capacity to provide informed responses (i.e., section VI of the survey), information about the current state of VR technology, efficacy evidence, uptake, side-effects, cost, logistics, billing, and a 2-min video demonstration of a clinical VR application to treat persecutory delusions was provided (see Supplementary Material; Figures 1A,B depicts screen-captures of the video shown to participants). The information section was reviewed by the research team for consensus around the accuracy and neutrality of information (i.e., not presenting information in a positively or negatively biasing manner) at the time of data collection.

*V. Impression of therapeutic VR after information provision*. This section asked participants to re-rate their overall impression of VR as a therapeutic tool in mental healthcare (“positive,” “neutral,” “negative”) after being provided with information about therapeutic VR. An optional short answer question was included to further explore participants' impressions.

*VI. Acceptability, Appropriateness and Feasibility*. Following provision of the information section, participants completed the implementation outcome measures, Acceptability of Intervention Measure (AIM), Appropriateness of Intervention Measure (IAM) and the Feasibility of Intervention Measure (FIM) (41). These were modified by substituting “evidence-based practice” with “therapeutic virtual reality.” Each scale consisted of four items rated on a 5-point Likert scale (1 = “strongly disagree” to 5 = “strongly agree”). A cut-off score of ≥ 15 indicated whether a participant perceived VR as acceptable, appropriate, or feasible to implement within their role or service setting. These scales have demonstrated good structural validity (Cronbach's alphas of 0.85 for AIM, 0.91 for IAM and 0.89 for FIM) and test-retest reliability (Cronbach's > of 0.83 for AIM, 0.87 for IAM and 0.88 for FIM). This section also included optional short answer questions to further explore participants' perceptions about the usefulness of therapeutic VR and potential barriers to its use within one's clinical role or service.

### Data Analysis

#### Quantitative

The datasets for analysis in this manuscript are available upon request, without reservations, to all researchers. Of the 97 recorded survey attempts, only 81 contained full datasets which were included in analyses. Analyses were conducted using *SPSS Statistics Version 22*. Descriptive statistics were used to summarize data related to demographics, knowledge and attitudes, and AIM, IAM and FIM total scores. Due to small, unequal sample sizes and indications of non-normality and negative skew on AIM, IAM and FIM scores through Shapiro-Wilks tests and visual analysis of graphs, non-parametric tests were conducted (42). Mann-Whitney non-parametric tests were used to explore differences on AIM, IAM, and FIM scores between clinical and non-clinical staff, participants who had or had not tried VR, and males and females. Spearman's rho correlations were performed between years worked in current or similar role and respective AIM, IAM, and FIM scores. All tests were two-tailed, with an alpha level of 0.05 (43).

#### Qualitative

Data was de-identified and initially divided into responses for each short answer question, which were subsequently considered as a whole during category development. Comments (*n* = 251) were analyzed with broad thematic grouping using a deductive approach informed by Proctor et al.'s (32) implementation outcomes. To provide further structure, responses were sorted into second-order codes based on stakeholder position (e.g., clinician, patient, service), then further classified as a barrier or facilitator. Barriers were factors that would prevent or hinder use, implementation, or uptake, while enablers were factors that would enhance or increase use, implementation, or uptake. Extraneous comments were excluded from further analysis. An individual response could contribute to more than one category if several issues were addressed. The number of comments reported in tables are the number of comments that contained information for each sub-category.

#### Data Integration

Data were collected using a sequential explanatory strategy (44), with quantitative data collection preceding free-text comments, which were used to contextualize and triangulate quantitative data on AIM, IAM, and FIM.

## Results

### Knowledge of VR

Prior to undertaking the survey, 91% (*n* = 74) of participants had heard of VR technology, and 42% (*n* = 34) of participants had previously tried VR at commercial gaming outlets (*n* = 15), residential homes (*n* = 10), museums, shopping centers, and theme parks (*n* = 8) or as part of a research study (*n* = 1). Of those who indicated they had not tried or were unsure if they had tried VR (*n* = 47), 92% indicated they would like to.

### Knowledge of Therapeutic VR

Prior to undertaking the survey, 40% (*n* = 32) of participants had heard of VR being used clinically in mental healthcare. None of the participants reported having used therapeutic VR with a patient.

### Attitudes Toward Therapeutic VR

Prior to reading the information section of the survey, 65% of participants had a positive impression and 36% had a neutral impression of VR being used in mental healthcare. After reading the information section, 84% of participants reported a positive impression, 14% reported a neutral impression, and 1% reported a negative impression (*n* = 1 missing). Seventeen participants (21%) changed their impression from neutral to positive after reading the information section, while two others (3%) changed their impression from positive to neutral or neutral to negative.

### Acceptability, Appropriateness, and Feasibility

Descriptive statistics for group comparisons on AIM, AIM, and FIM scores are presented in Table 4. After reading the information section of the survey, 84% of participants felt that VR was acceptable, 69% of participants felt that VR was appropriate, and 59% of participants felt that VR was feasible to introduce into private mental health services or their clinical role. Clinicians perceived VR to be less appropriate, *z*_(N = 81)_ = 2.15, *p* = 0.03, *r* = 0.24, and less feasible, z_(N = 81)_ = 2.15, *p* = 0.03, *r* = 0.24, to implement than non-clinical staff, however, there was no difference in acceptability scores, *z*_(N = 81)_ = 0.17, *p* = 0.86, *r* = 0.02. Hospital workers who had previously tried VR perceived it to be more acceptable, *z*_(N = 77)_ = 1.96, *p* = 0.02, *r* = 0.22, and more appropriate, *z*_(N = 77)_ = 2.46, *p* = 0.005, *r* = 0.28, than those who had not tried VR, however there was no statistically significant difference in feasibility scores, *z*_(N = 77)_ =1.56, *p* = 0.12, *r* = 0.18. There were no statistically significant differences between males and females in scores for acceptability, *z*_(N = 80)_ = 1.93, *p* = 0.05, *r* = 0.22, appropriateness, *z*_(N = 80)_ = 0.07, *p* = 0.95, *r* = 0.01, or feasibility, *z*_(N = 80)_ = 0.38, *p* = 0.70, *r* = 0.04. Spearman correlations conducted between years worked in current or similar role and scores for acceptability, *r*_*s*_ = 0.01, *p* = 0.99 appropriateness, *r*_*s*_ = −0.039, *p* = 0.73 and feasibility, *r*_*s*_ = −0.0454, *p* = 0.69, were not statistically significant.

**Table 4:** Descriptive statistics and group comparisons on IAM, AIM, and FIM.

		**Acceptability (AIM)**		**Appropriateness (IAM)**		**Feasibility (FIM)**	
	***n***	**Median (25th;75th)**	**Min-Max**	**Median (25th;75th)**	**Min-Max**	**Median (25th;75th)**	**Min-Max**
Clinical	52	17.50 (16; 20)	12–20	16.00 (12; 18.25)	4_20	14.00 (12; 16)	4–20
Psychiatrists	7	20.00 (16; 20)	13–20	20.00 (16; 20)	12–20	20.00 (16; 20)	12–20
Psychologists	9	16.00 (13; 18)	12–20	14.00 (10; 16)	9–16	13.00 (12; 14)	4–16
Nurses	25	20.00 (16; 20)	12–20	16.00 (13.5; 20)	8–20	16.00 (14; 18)	11–20
Other allied health	11	16.00 (15; 17)	12–20	12.00 (12; 16)	4–16	12.00 (11; 13)	4–16
Non-clinical	29	19.00 (14.5; 20.00)	12–20	16.00 (16; 20)	10–20	16.00 (15; 19)	12–20
Management	9	16.00 (16; 20)	14–20	20.00 (14.5; 20)	12–20	16.00 (15; 19.5)	14–20
Administration	14	16.00 (13; 20)	12–20	16.00 (15; 18.5)	10–20	16.00 (12; 19.25)	12–20
Research/student	6	17.50 (15; 20)	15–20	18.00 (16; 20)	16–20	15.50 (14.75; 18.25)	14–19
Tried VR	34	20.00 (16; 20)	13–20	16.00 (16; 20)	10–20	16.00 (14; 18.25)	12–20
Not tried VR	43	16.00 (15; 20)	12–20	16.00 (12; 16)	4–20	15.00 (12; 19)	4–20
Male	22	20.00 (16; 20)	13–20	16.00 (13; 20)	4–20	15.00 (14; 18.25)	12–20
Female	58	16.00 (15; 20)	12–20	16.00 (12; 20)	8–20	16.00 (12; 18.25)	4–20
Total	81	18.03 (16; 20)	12–20	16.00 (12.5; 20)	4–20	16.00 (12.5; 18)	4–20
Clinical vs. non-clinical	81	*p* = 0.864		*p* = 0.031^*^		*p* = 0.031^*^	
Tried VR vs. not tried VR^a^	77	*p* = 0.019^*^		*p* = 0.005^*^		*p* = 0.118	
Male vs. female^b^	80	*p* = 0.054		*p* = 0.946		*p* = 0.703	

*Results are presented as median and 25th (Q1) and 7th (Q3) percentiles. p-values for between-group comparisons with Mann-Whitney U-tests.*

* *p < 0.05.*

a *Participants who reported being unsure if they have tried VR (n = 4) were excluded from analyses.*

b *One participant did not report gender*.

### Barriers and Enablers to Implementation

Among free-text comments, 80% were related to acceptability, 44% were related to appropriateness, and 32% were related to feasibility. The most common barriers and enablers reported for each category are outlined in Table 5.

**Table 5:** Perceived implementation barriers and enablers organized by stakeholder level.

**Category**	**Stakeholder**	**Comments** ***n* (%)**	**Barriers**	**Enablers**
Acceptability	Clinician	155	Perceived technical difficulties/ limitations of VR Lack of knowledge/experience with VR Belief about negative outcomes from VR use Discomfort/disinterest with new technology	Perceived clinical and/or practical benefits Positive attitudes for technology/changes to practice Knowledge about clinical VR applications Prior/future opportunities to experience VR VR perceived as enjoyable and/or easy to use
	Patient	37	Patient discomfort with new technology Patient lacking readiness for treatment Limited awareness/understanding of therapeutic VR	Belief VR will enhance engagement/ help-seeking Provide education and trial VR with patients Increased treatment choices
	Service	3	Lack of vision or will to introduce VR	Positive feedback from staff and patients
Appropriateness	Clinician	93	Concerns about clinical risk and safety Incompatible with treatment philosophy	Perceived suitability for specific disorder/treatment Belief VR is safe to use
	Patient	10	Lack of ability to engage (e.g., chronicity, severity)	Belief VR is helpful for learning/practicing skills
	Service	5		Fits with scope of treatments offered by service Helpful for clinical training purposes
Feasibility	Clinician	32	Logistical setting constraints (e.g., groups, outreach) Lack of skills/training Lack of time to use VR	Access to training and resources
	Patient		Lack of access to VR (e.g., rural patients)	
	Service	42	Lack of funding and available resources Lack of clinical expertise/governance Lack of service reimbursement	Planning and clinical support/governance VR perceived as cost-effective Perceived introduction strategy (e.g., research trial)

#### Acceptability

##### Barriers

The most common barrier to acceptability related to perceived technical difficulties and limitations of VR. Participants anticipated limitations in VR, including the “reliability of equipment” (P07, Psychiatrist) and “customisability of software to meet the patient's individual needs/fears” (P70, Researcher). Participants expressed concerns about VR being an “artificial environment… similar to a game and not actually getting on the train or in the lift” (P39, Clinical Manager), thus preventing gains made in VR transferring into real-world improvements. Staff also queried whether clinical VR research could keep pace with the rapidly evolving technology [e.g., *technology rapidly evolving but may mean is unable to be empirically tested in a considered way* (P33, Researcher)]. Other barriers related to staff “not ever experiencing VR” (P10, Nurse) or a “lack of knowledge” (P3, Program Manager) about the efficacy of therapeutic VR and recommended practice (e.g., contraindications, treatment timing, dosage, after-care). Participants also expressed concerns that private health providers might look to “replace service provision with virtual reality” (P20, Intake Coordinator) and that VR might replace “human interactions and connections” (P53, Psychologist). A lack of “willingness to try” (P10, Nurse) VR due to factors including age and discomfort with technology was another perceived barrier for use, with participants anticipating that “older patients may not embrace the technology” (P16, Organizational Administrator) and that it “may only appeal to younger or tech savvy patients” (P68, Student).

##### Enablers

Perceived clinical and practical benefits of VR were the most common enablers to acceptability. Participants viewed VR as “another tool in the toolbox” (P78, Service Manager), with potential to “make intervention quicker [due to] opportunity to practice [skills] in a realistic setting before moving into real life” (P39, Clinical Manager). Participants perceived benefits in being able to access “environments… otherwise difficult to access” (P4, Program Manager), managing “difficult scenarios with low risk” (P45, Nurse), making treatment “less reliant on the quirks of humans” (P39, Clinical Manager), and enhancing control in exposure therapy (e.g., “if it becomes overwhelming, is easily removed and stopped” (P16, Organizational Administrator). Other enablers related to positive attitudes about technology-use in clinical practice (e.g., “it is the way the future is heading to be implementing more technology into therapy,” [P47, Psychologist]), and perceptions that it could “reach people who have difficulty engaging in treatment services in typical ways” (P33, Researcher). Participants felt VR would “appeal to a younger market [and] different cohort of patients” (P56, Program Manager), and benefit patients who had “difficulty articulating their experiences, [are] experientially avoidant” (P33, Researcher), or who “lack capacity to organize themselves” to complete exposure tasks when at home” (P64, Occupational Therapist). Prior experiences with VR enhanced participants' understanding of its therapeutic potential [e.g., “During the game…dealing with heights, of which I'm afraid…this was affected” (P15, Nurse)], while knowledge of its clinical applications enhanced its credibility [e.g., “Barbara Rothbaum at Emory in USA is already using this to treat PTSD.” (P09, Psychiatrist)]. Broader dissemination of “impartial… findings” (P20, Intake Coordinator) particularly around efficacy, and allowing staff to” try and experience the feeling of VR” (P35, Nurse) were strategies suggested to further enhance acceptability.

#### Appropriateness

##### Barriers

Among barriers to appropriateness, most comments related to concerns around clinical risk and safety. Participants expressed concerns that VR would be used “without adequate training…[or] proper assessment of the client” (P26, Psychologist), which could “exacerbate symptoms” (P27, Psychologist), or lead to “avoidance of the real world” (P15, Nurse). Participants were also concerned about “potential unknown adverse reactions… [in patients with] a history of dissociation, derealization” (P33, Researcher), “severe PTSD and paranoia” (P18, Nurse), “psychosis” (P22, Program Manager) as well as “adverse reactions with medication” (P16, Organizational Administrator). Participants wondered “how patients with auditory hallucinations would cope” (P48, Nurse) and worried that patients may become “too overwhelmed from what they are seeing…and not speak up” (P60, Nurse) resulting in patients or staff injury or equipment damage. Participants also worried about immersion side effects including “disorientation and nausea [based on] personal experience” (P32, Psychiatric Nurse) and “electrical cords exposed [presenting a] hanging risk” (P69, Researcher). Other barriers related to perceived incompatibility with one's treatment philosophy (e.g., “Art therapy seeks to stimulate creativity and 'play'. It is strength based and virtual reality would not be of assistance” [P30, Art Therapist]).

##### Enablers

The most common enablers to appropriateness were perceptions that therapeutic VR would be “safe for clients” (P49, Psychologist) and was suitable for the scope of clinical presentations encountered and treatments offered by their service [e.g., “great opportunity…not unlike offering ACT, DBT and TMS.” (P65, Service Manager)]. Participants perceived VR as having “many applications across diagnostic groups” (P76, Program Manager), including anxiety disorders, PTSD, OCD, and addictions. Staff also felt that it was compatible with a range of interventions, including “graded exposure” (P40, Occupational Therapist), “role play” (P15, Nurse), “mindfulness” (P20, Intake Coordinator), “challeng[ing]…maladaptive beliefs” (P62, Nurse), “relaxation…to manage stress” (P23, Nurse), “biofeedback” (P64, Occupational Therapist), as well as relapse management [e.g., “safe way of simulating exposure to risk situations without the problems of relapse” (P39, Clinical Manager)] and harm minimization [e.g., “helping patients deal with suicidal or self-harm thoughts without actually doing these acts.” (P18, Nurse)]. Participants also felt that VR could help in the functional recovery of patients requiring “a slow return to community/social exposure” (P35, Nurse) and to “step up and/or step down for admissions to hospital” (P33, Researcher). Another facilitator was perceived reputational benefits, which could help position their service as a specialist clinic [e.g., “Would be a great adjunct to the PTSD program…and further [our] name as center of excellence in PTSD.” (P09, Psychiatrist)].

#### Feasibility

##### Barriers

A major barrier to feasibility related to logistical constraints of service settings. Participants noted that within the psychiatric hospital setting, clinicians “mainly work in group” (P39, Clinical Manager), and perceived VR to be an “isolating activity” (P31, Exercise Physiologist) that would be practically difficult to incorporate into group sessions [e.g., “couldn't take time to (use VR) with each individual while others waited” (P57, Psychologist)]. Participants anticipated challenges to obtaining adequate funding “for 1:1 work alongside group work” [P64, Occupational Therapist], to purchase and maintain “multiple units” (P31, Exercise Physiologist), and ensuring that content would be “appropriate for all group members” (P31, Exercise Physiologist). Other common barriers related to a lack of expertise to provide “adequate training for clinicians” (P62, Nurse), “time required to train and use [VR and having] limited physical space” (P38, Psychologist) and the “portability/ transport of equipment and set up in outreach” (P52, Nurse). Participants also perceived that VR may not be viewed as financially lucrative enough to prioritize investing in by the service's private owners given its lack of service reimbursement [e.g., “I am concerned about how it is billed and therefore financially lucrative for the private health sector.” (P56, Program Manager)].

##### Enablers

The most common enablers to feasibility related to the availability of VR “headsets [and] proper training” (P20, Intake Coordinator) for staff to deliver it. Participants felt that barriers related to “funding, space, resources [could] be overcome with planning” (P56, Program Manager), however, noted that clinical consultation was needed and that “psychologists or psychiatrists [should] be responsible for at least overseeing VR implementation.” (P10, Nurse). Participants suggested that “private health non-inclusion” could be overcome by offering VR as “part of therapy in a structured admission” (P63, Service Manager). Introducing therapeutic VR through a research trial was also seen as facilitatory, as had been previously done with other recently introduced treatments to services [e.g., “It is a great opportunity to do a research trial much like when TMS was commenced.” (P63, Service Manager)].

## Discussion

In light of recent commercial availability of affordable VR hardware and the demonstrated efficacy of numerous therapeutic VR environments, mental health providers have an opportunity to capitalize on the potential benefits of this immersive technology. Understanding the perceived acceptability, appropriateness, and feasibility of implementing therapeutic VR among frontline stakeholders is a fundamental step to ensuring its timely, effective, and sustained uptake in clinical settings. Overall, findings demonstrated a strong interest in therapeutic VR among Australian private mental health service staff, despite low familiarity with the technology and its clinical applications. The majority (84%) of respondents had a positive impression of VR after provision of general information, most (84%) staff reported that therapeutic VR was acceptable, and over half indicated it was appropriate (69%) and feasible (59%) to implement within their role or service. These findings suggest that the potential benefits of VR are largely self-evident to staff, but that limited awareness of the clinical evidence-base and available systems to integrate into current treatment and service models remain key barriers to uptake.

Findings suggest value in broad stakeholder engagement, as clinicians perceived VR to be less appropriate and feasible to implement than non-clinical service staff. This may reflect possible tension between managers, who typically decide whether new treatments are practical to introduce and oversee their integration into service operations, and clinicians, who refer patients for and deliver treatment. Addressing clinicians' reservations will be important as research suggests that negative attitudes have a stronger influence on future VR use (i.e., non-use) among CBT therapists than positive attitudes (28), and that failure to shift negative beliefs can adversely impact treatment availability and quality (45). Notably, psychologists and allied health professionals found VR least appropriate and feasible to implement. Thus, differentially targeting these disciplines during implementation efforts could be useful, for instance, by incorporating VR into graduate training programs, as has occurred in surgical and nursing education (46, 47).

Among the most common barriers staff reported were perceived risk and safety issues. This suggests that a major focus of education and training should involve addressing negative preconceptions about patient suitability (e.g., contraindications) and providing guidance around the likelihood and management of potential adverse events (e.g., patient distress, side-effects, medication interaction). Inaccurate beliefs about treatment consequences (e.g., iatrogenic effects, reduced therapeutic alliance) have been a well-documented barrier to the dissemination of *in vivo* exposure therapy, which remains underutilized even among trained clinicians (45). Current evidence suggests that concurrent use of common pharmacological treatments (e.g., olanzapine, antipsychotics, antidepressants) with VR have minimal adverse effects (20, 23, 48, 49) and that alliance when using VR is similar to face-to-face therapy (50, 51). However, both areas remain under-researched and warrant further attention. Interestingly, information about a VR application to treat persecutory delusions did not completely allay participants' concerns about its appropriateness for psychosis treatment, suggesting that knowledge alone may be insufficient to change attitudes.

Another common barrier reflected perceived technical challenges and therapeutic limitations (e.g., low customisability, real-world generalisability), consistent with barriers identified in previous research (28, 34, 35). These unfavorable perceptions are likely attributable to low familiarity with VR technology generally, as participants with prior VR experiences perceived it to be more acceptable and appropriate to implement in mental healthcare than those without. Segal et al. (35) similarly found that therapists with greater knowledge or interest in VR perceived it as having greater clinical utility. A lack of experience with immersive VR technology or experiences exclusively in recreational contexts could negatively skew perceptions about its clinical utility by underselling the sophistication and quality of purpose-built therapeutic environments optimized for high-fidelity HMDs. Indeed, more advanced immersive VR technology has been associated with higher levels of presence and subjective anxiety (52), which has important implications for exposure therapy, whereby corrective emotional processing requires sufficient activation of one's fear structure (53). These concerns will likely diminish as VR becomes more commonplace across society (e.g., entertainment, education, medicine), and as results of clinical research are more widely disseminated. For instance, meta-analytic findings suggest that gains made through VRET for specific phobias transfer to the real-world (52, 54), though further investigation in other psychiatric conditions is required to substantiate efficacy. Similarly, customizability of therapeutic VR programs (i.e., grading virtual scenes for increasing degrees of exposure difficulty) is becoming increasingly common (55, 56).

Encouragingly, staff perceived VR as having broad applicability across mental health conditions, interventions, and assessments. These findings are consistent with Lindner et al. (28), highlighting greater scope for collaboration between VR developers and clinical stakeholders to develop applications beyond exposure therapy (e.g., relapse prevention, harm minimization). Greater stakeholder consultation during application development may also help maximize its utility in applied service contexts and address practical challenges across service settings. For instance, staff questioned the feasibility of VR in group therapy, which was identified as a predominant treatment delivery mode. While current therapeutic applications offer limited solutions to this, advancements in multi-user VR systems may yet open possibilities for immersive group therapy in the future (57).

Staff also anticipated challenges to adequate resourcing (e.g., trained staff, rooms, purchasing, and maintaining multiple HMDs). Given that VR-based therapies have yet to demonstrate superiority over current treatment approaches (58, 59), and that few products are being reimbursed as a specific treatment by private health funds, highlighting VR's potential to address existing challenges in clinical practice may be a useful strategy to promote uptake. For instance, research suggests that consumers perceive VRET as more acceptable than *in vivo* exposure and that it can enhance motivation and engagement with treatment (17, 60). Evidence of cost-effectiveness could also be an incentive, as has been shown with psychosis and combat PTSD populations (61, 62). Additionally, automated VR treatments have shown early promising results for acrophobia and psychosis (15, 28) and may offer a low-cost strategy to scale effective intervention (3). Nonetheless, participants' concerns about VR replacing service provision suggest that automated applications could risk enhancing unfavorable perceptions of VR, thus further research on their efficacy and safety and careful attention during their dissemination is warranted.

Our findings have practical implications for the implementation of VR applications in mental healthcare. Participants limited knowledge of VR and its therapeutic applications speaks to a need for greater education and training, which could be delivered through graduate training programs, e-learning modules, and interactive workshops. The lack of resources to guide implementation has been identified as a barrier to the use of technology-enabled treatments more broadly (3). Thus, there is a need to develop evidence-based practice guidelines to ensure safe and ethical usage of evidence-based therapeutic VR applications.

It is important to note that while commercially available products (see Table 2) are developed with evidence-based principles in mind, most have not undergone, or are in the process of being tested for efficacy in randomized controlled trials (29). Similarly, freely available VR experiences are often marketed as having a mental health focus on open application marketplaces (e.g., SteamVR, Oculus), without verification of clinical utility or safety (63). Thus, there is a risk of unvalidated VR programs being marketed for both therapist-assisted or self-directed therapy, with potential to cause harm to users and diminish the credibility of validated programs (9). Nonetheless, evidence-based interventions (e.g., exposure therapy, arousal reduction, behavioral activation) in routine care often involve use of imaginal, visual, or auditory stimuli to elicit target emotional states (e.g., anxiety, disgust, craving, enjoyment, relaxation), for which the clinician and patient will often creatively source. Thus, VR scenarios will at least have value as an adjunct to these interventions. In future, we may see greater involvement of regulatory bodies in the dissemination of digital therapeutic, including VR therapies, as administrations in the US, EU, UK, and Australia, have or are in the process of reforming their regulatory framework for therapeutic software in recognition of their growing ubiquity and potential risk profile (64).

From a technical standpoint, the rapid pace of VR development also presents challenges with synchronization and compatibility of software across systems (59). Moreover, the literature to date has suffered from ambiguous terminology, leading to inadequate specification and misclassification of VR, which could diminish literature validity and provider confidence in therapeutic VR. Thus, as the field progresses, it stands to benefit from greater standardization of VR, for instance, with Takac et al.'s (65) proposed hardware-based VR qualification matrix, as well as development of a therapeutic VR resource directory. A similar initiative funded by the Australian government (i.e., e-Mental Health in Practice) successfully raised awareness of evidence-based digital mental health interventions among primary healthcare providers. Thus, these resources will likely be critical to enhancing providers' “technological competence” in selecting and recommending appropriate VR hardware.

The current study adds to the literature by documenting the knowledge, attitudes, and perceived implementation barriers for therapeutic VR use among a broader stakeholder group (i.e., clinical, managerial, and operational staff perspectives), and including validated implementation outcome measures. However, the results should be considered with some caveats in mind. Limitations include the modest sample size and specificity to the Australian private psychiatry setting providing services to a predominantly adult patient population, which may limit generalisability. Another limitation related to participants' low familiarity with VR, necessitating provision of general information to encourage informed responses. Nonetheless, given the limited uptake of VR in mainstream clinical settings across Australia and other comparable countries (e.g., US, UK), these findings are likely representative of current knowledge and diffusion of VR in private psychiatric services, and will therefore be relevant to early dissemination efforts in similar settings. An important next step that future research should address will be to document the perspectives of staff from public healthcare services, those working with pediatric and adolescent patient groups, and critically, those of consumers of mental health services.

We conclude that while there is a clear appetite for VR among Australian mental health providers working in the private system, concerns related to safety, efficacy, and logistical barriers warrant attention. Addressing unhelpful beliefs and knowledge and skills gaps will help ensure that prospective providers are adequately informed and empowered to adopt VR into clinical practice. Further research focused on formulating and evaluating implementation strategies is needed to promote effective and sustained uptake.

## Data Availability Statement

The raw data supporting the conclusions of this article will be made available by the authors, without undue reservation.

## Ethics Statement

The studies involving human participants were reviewed and approved by Melbourne Clinic Human Research Ethics Committee (#304) and the Monash University Human Research Ethics Committee (#13284). Written informed consent was not provided because informed consent was implied through survey completion.

## Author Contributions

AJ and RS designed the study. AJ and ND collected the data. OC analyzed the data and drafted the manuscript. OC, ND, TR, CN, MY, and RS critically reviewed the data interpretations and draft and made significant contributions to the final version. All authors contributed to the article and approved the submitted version.

## Funding

This work was supported by philanthropic investment from The David Winston Turner Endowment Fund, Wilson Foundation. The funding sources had no role in the design, management, data analysis, interpretation, and write-up of the data.

## Conflict of Interest

The authors declare that the research was conducted in the absence of any commercial or financial relationships that could be construed as a potential conflict of interest.

## Publisher's Note

All claims expressed in this article are solely those of the authors and do not necessarily represent those of their affiliated organizations, or those of the publisher, the editors and the reviewers. Any product that may be evaluated in this article, or claim that may be made by its manufacturer, is not guaranteed or endorsed by the publisher.
