# Supplementary material for: Are Australian Mental Health Services Ready for Therapeutic Virtual Reality? An Investigation of Knowledge, Attitudes, Implementation Barriers and Enablers
Source: Front Psychiatry. 2022 Feb 4;13:792663. doi: 10.3389/fpsyt.2022.792663 (PMC8854652; doi:10.3389/fpsyt.2022.792663)
Supplement: Supplementary file 1 [file Presentation_1.PDF]

MONASH University

The Melbourne Clinic

Explanatory Statement

Therapeutic Virtual Reality: Implementing the next frontier of technology into clinical practice

What is this study about?

Rapid advances in virtual reality technologies have created the means to immerse people in safe, realistic, personalised, therapeutic environments. In virtual reality people can face their fears, work through their anxieties, and attempt to re-train new response to their personal clinical triggers, all without leaving the clinicians office. Evidence for the efficacy of therapeutic virtual reality in treatment of a range of mental illnesses is growing rapidly.

A key challenge, however, will be the implementation of virtual reality into clinical health care settings. Historically, only 50% of new evidenced based practices make it from research environments to the clinic. Of those that do, the average time to translation is 17 years from discovery, and traditionally mental health care services have been particularly slow to adopt new technologies.

This study aims to document knowledge and opinions of staff at a private mental health care service about therapeutic virtual reality and to identify potential benefits and barriers to its implementation into clinical practice.

Please note

There is no plan to introduce therapeutic virtual reality into clinical practice at The Melbourne Clinic in the near future.

Rather, The Melbourne Clinic is an exemplar of a high-quality private mental health service and internationally many such services are currently, or will soon be, seeking to implement therapeutic virtual reality. We are asking you to share your knowledge and opinions about therapeutic virtual reality as a means of understanding representative issues that could be helpful for a wider audience of mental health care services and therapeutic virtual reality developers.

What does this study involve?

This anonymous survey comprises a short series of multiple choice and (optional) short answer questions about:
a) your demographics and job role,
b) your knowledge of and experience with virtual reality,
c) your thoughts about the usefulness and feasibility of introducing therapeutic virtual reality into private mental health care services.

In the middle of the survey you will also find some brief information about therapeutic virtual reality and a short (2-minute) video illustrating an example of its use.

How long will it take?

The survey will take 8 – 10 minutes to complete.

Why have I been invited to take part?

As a member of staff or student at The Melbourne Clinic you may have professional knowledge or experience in administering therapeutic interventions, and/or an understanding of the day-to-day operations and management of a private mental healthcare service.

It is our hope that your responses will help identify whether therapeutic virtual reality might be useful to you, your patients, your service, and other services like yours around the world, and, if so, discover how best to introduce it into clinical practice.

What are the risks and benefits?
This survey is anonymous, confidential, and does not pose any risks.

We greatly appreciate your sharing your knowledge and opinions. If you choose to enter your name email address the end of the survey you will go into the draw to win a $200 Coles Myer voucher. Your contact details will not be linked to your survey responses.

Do I have to take part, and how will my responses be handled?
Participation in the survey is entirely voluntary and completion of this survey implies consent. You may withdraw from participation at any stage prior to submitting your responses without further implication or contact from the research team. Information collected will be stored securely in accordance with The Melbourne Clinic and Monash University ethics committee regulations, and no personally identifying information will be collected.

Who do I ask for further information?
If you would like further information, please contact the lead investigators.

Therapeutic Virtual Reality Survey

Question Colour Key

Questions for all respondents (No colour).
Questions for respondents with a clinical role.
Questions for respondents with a non-clinical administration or managerial role.

I – Demographic Questions

1. **What is your gender?**
   - Female
   - Male
   - Other
   - Prefer not to say
2. **How old are you?**
   - 18, 19, 20…. 70+. (drop down list).
   - Prefer not to say
3. **What is your primary role at The Melbourne Clinic?**
   - Clinical care of patients
   - Administrative
   - Managerial
   - Student
   - Other (please specify)

   a.) (if clinical)

   - Psychiatrist
   - Psychiatric registrar
   - Psychologist
   - Psychology registrar
   - General Practitioner
   - Nurse
   - Occupational Therapist
   - Social Worker
   - Physiotherapist
   - Student, please specify area of training ____________
   - Other, please specify _________

   b.) **(if clinical) Does your role involves delivery of psychological therapies with patients**
   - Yes
   - No

   c.) **(if yes to therapy) Thinking about your typical therapeutic work, on average, what percentage of group therapy and individual therapy do you deliver?**
   - % working with group therapy
   - % working with individual therapy

   d.) **(if clinical) Thinking about your typical therapeutic work, on average, what percentage do you working with the following patient groups:**
   - % patients aged 16 to 24 years (i.e. child and adolescent)
   - % patients aged 25 to 64 years (i.e. adults)
   - % patients aged 65 years and above (i.e. old age / geriatrics)

***e.) If* Administrative**

- Reception
- Organisational / operational
- Financial
- Other, please specify __________.

***f.) If* Manager**

- Unit manager
- Program manager
- Office manager
- General manager
- Other, please specify __________.

***g.) If* Student**

- Please specify area of training ______________.

4. How many years have you been working in this role or a similar role?

- (drop down list) 1 year or less, 2 years…. 30+ years.

5. Thinking about your typical working week, what percentage of time do you spend working with inpatients versus outpatients?

- % working with inpatient setting
- % working with day program setting
- % working with outreach setting

Must total 100%

Must total 100%

6. Thinking about your therapeutic work over a typical working week, on average <u>what percentage of time</u> do you spend working with group therapy versus individual therapy?

- % working with group therapy
- % working with individual therapy
- My work does not involve delivering therapy

7. Thinking about your typical working week, on average what are <u>the three primary diagnoses</u> you spend the most time working with? Please list these in descending order, i.e. 1 = I spend the most time working with patients for whom this is their primary diagnosis.

- Major Depression
- Bipolar Affective Disorder
- Obsessive Compulsive Disorder
- Anxiety Disorders (excluding OCD)
- Post-Traumatic Stress Disorder
- Addictions
- Eating Disorders
- Personality Disorders
- Psychotic disorders
- ADHD
- Neurological Disorders (Alzheimer’s, Delirium)
- Other, please specify

II – Knowledge of Virtual Reality

The following questions ask about your current knowledge and exposure to virtual reality before taking this survey. Its fine if you haven't heard much about virtual reality, or used it, it's helpful for us to document this also.
These questions ask about virtual reality in general and are not asking about your thoughts or exposure to therapeutic virtual reality. We ask those in the next section.

1. **Before taking this survey, had you heard of virtual reality?**
    - o Yes
    - o No
    - o Not sure
2. **Have you ever used virtual reality?**
    - o Yes
    - o No
    - o Not Sure

    a.) (*If yes*) **Where did you try virtual reality?**
    - o At home, I have my own virtual reality
    - o At a friend or family members house - they have their own virtual reality
    - o At a commercial virtual reality games outlet
    - o At a museum
    - o Other, please describe: _____________

    b.) *(If no)* **Would you like to try virtual reality?**
    - o Yes
    - o No
    - o Not Sure
3. **Based on what you know or have heard about virtual reality (even if it's not very much), which of the following best describes your impression of it:**
    - o I have a positive impression of virtual reality
    - o I have a negative impression of virtual reality
    - o I have no impression / a neutral impression of virtual reality
4. (*Optional short answer*) **We would be grateful if you'd share one or more of the reasons for your impression.**

III – Knowledge and impression of Therapeutic Virtual Reality

The following questions relate specifically to the use of virtual reality as a therapeutic tool.

1. **Before this survey, had you heard of virtual reality being used therapeutically in medicine (i.e. for areas of health other than mental health care)?**
   - Yes
   - No
2. **Before this survey, had you heard of virtual reality being used therapeutically in psychology or psychiatry (i.e. specifically for mental health care)?**
   - Yes
   - No
3. **Have you ever used therapeutic virtual reality with patients?**
   - Yes
   - No
4. **What is your impression of virtual reality being used as a therapeutic tool for mental health care?**
   - I have a positive impression of therapeutic virtual reality for mental health care
   - I have a negative impression of therapeutic virtual reality for mental health care
   - I have no impression / neutral impression of therapeutic virtual reality for mental health care
5. **(*Optional short answer*) We would be grateful if you'd share one or more of the reasons for your impression.**

IV – Information Section about Virtual Reality

Immersive virtual reality involves putting on a headset which allows the user to view and interact with a 3-dimentional computer generated virtual environment. As the user moves around the real world, either by turning their head or walking, they experience this as moving around the virtual world. In virtual reality people can experience simulated sight, sound, touch, and balance.

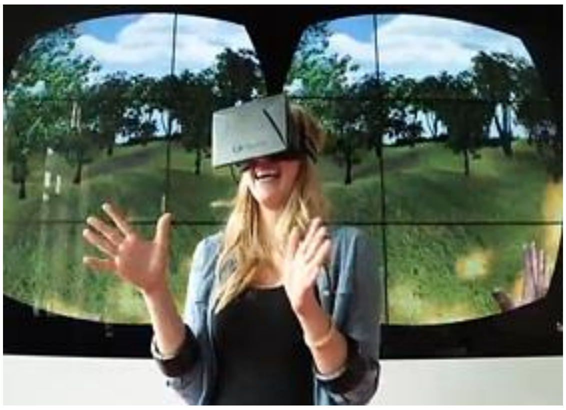

What is Therapeutic Virtual Reality?

Therapeutic virtual reality involves immersing people in simulations of scenarios that are relevant for their psychological difficulties. Examples include, an airplane cabin for someone with fear of flying, a casino for someone with gambling addiction, or a conflict zone for military personal experience PTSD.

The goal is to allow people to experience their personal clinical triggers within a realistic but safe environment, and for them to receive clinical coaching that helps them learn to think, react and behave in more adaptive ways. Virtual environments can be graded in difficulty and repeatedly experienced, without leaving the therapist office.

This two-minute video illustrates an example of therapeutic virtual reality, please watch it and read the information below before completing the remaining survey questions.

<insert VR Paranoia Video by Oxford University here: https://youtu.be/YhbkELUSzqQ>

Evidence for therapeutic benefits

- Clinical research into therapeutic virtual reality is increasing rapidly, with the number of published studies up from 1 in 1985 to 945 studies in 2017.

- Many small or uncontrolled research studies have demonstrated therapeutic benefits of therapeutic virtual reality, but only a small number of high quality randomised controlled trials have been conducted.

- Currently, the best evidence for therapeutic virtual reality is in exposure-based therapies for anxiety disorders, particularly specific phobias, social phobia, and PTSD. Both randomised controlled trials and meta-analyses have showed strong evidence of clinical benefit for these conditions (Reger et al, 2016, J Consult Clin Psychol; Bouchard et al, 2016, Br J Psychiatry; Opris et al, 2012, Dep Anx).

- Other therapeutic applications in development include eating disorders, substance addictions, gambling addiction, depression, autism, and psychotic disorders. These indications have been less studied and the current evidence is positive, but preliminary (Freeman et al, 2017, Psychol Med).

- A research study investigating exposure and response prevention therapy for people with contamination-related OCD and comparing symptom provocation in response to real-world versus virtual reality delivered therapy is currently underway at The Melbourne Clinic.

Uptake around the world

- Therapeutic virtual reality is being offered in a small number of private mental health care services in the UK, USA, India, Spain, Ireland, Israel, Belgium, China, and some parts of Australia.

- In February 2018 the UK National Health Service (NHS) invested £4 million in the development of virtual reality therapy treatment trials and their implementation across the NHS network.

Side effects

- For a minority of people virtual reality can induce motion-sickness like symptoms, including nausea, headache, disorientation, sweating, and general discomfort.

  This is thought to be caused by a brief time lag between users movement in the real world (e.g. turning one’s head) and corresponding sensory perception in the virtual environment (e.g. visual scanning across a virtual room).

- Risk can be minimise by using a computer with very high processing capacity capable of running virtual environments at high speed, and by screening for susceptibility to motion sickness.

- It has been speculated that for some mental health patients spending time in virtual reality may exacerbate the symptom severity. It is unclear how likely a risk this is, and for whom it may be most relevant.

Cost and logistics

- The cost of virtual reality equipment (i.e. head set, accessories, high speed computer) is currently ~$3,000 - $4,000 AUD per unit.

  This is more than 200% cheaper than the cost on the early 2000's, and the price has been falling annually with rapid technological development and an expanding commercial market.

- The most immersive (i.e. realistic) forms of virtual reality allow the user to walk around a room in the real work and experience this as walking around the virtual environment. They require a medium sized room (approx. 6m by 6m) to run.

- The most common virtual reality technologies involve the patient entering the virtual environment alone, and the therapist viewing their experience on a computer monitor and communicating with them from outside the virtual environment. As such, it is better suited to individual therapy than group therapy.

Billing

- There is no Medicare or private health insurance rebate for any therapeutic virtual reality tools, and it is unlikely this will change in the near future.

- Some health services bill for access to their service (e.g. via a Mental Health Care Plan, or a private hospital admission) and offer therapeutic virtual reality as part of clinical care within their service.

V – Impression of Therapeutic Virtual Reality (after information provision)

Having seen and heard a little more about therapeutic virtual reality has your impression of it changed? Please re-answer the following question:

1. **What is your impression of virtual reality being used as a therapeutic tool for mental health care?**
   - I have a positive impression of therapeutic virtual reality for mental health care
   - I have a negative impression of therapeutic virtual reality for mental health care
   - I have no impression / neutral impression of therapeutic virtual reality for mental health care

VI – Acceptability, Appropriateness, and Feasibility of Therapeutic Virtual Reality

Acceptability of Therapeutic Virtual Reality

The following statements ask about how acceptable you think the implementation of therapeutic virtual reality in mental health care is.

1. **Using therapeutic virtual reality in mental health care meets my approval**
   - Strongly disagree
   - Disagree
   - Undecided
   - Agree
   - Strongly agree
2. **I welcome the use of therapeutic virtual reality in mental health care**
   - Strongly disagree
   - Disagree
   - Undecided
   - Agree
   - Strongly agree
3. **Use of therapeutic virtual reality in mental health care is appealing to me**
   - Strongly disagree
   - Disagree
   - Undecided
   - Agree
   - Strongly agree
4. **I like the idea of therapeutic virtual reality being used in mental health care**
   - Strongly disagree
   - Disagree
   - Undecided
   - Agree
   - Strongly agree

Appropriateness of Therapeutic Virtual Reality (Clinical Role)

The following statements are about how appropriate you think the implementation of therapeutic virtual reality in your clinical role is.

1. **Therapeutic virtual reality seems a fitting tool to use in my clinical role**
   - Strongly disagree
   - Disagree
   - Undecided
   - Agree
   - Strongly agree
2. **Therapeutic virtual reality therapy seems suitable to use in my clinical role**
   - Strongly disagree
   - Disagree
   - Undecided
   - Agree
   - Strongly agree
3. **Therapeutic virtual reality seems applicable to use in my clinical role**

- Strongly disagree
- Disagree
- Undecided
- Agree
- Strongly agree

4. Therapeutic virtual reality therapy seems like a good match for my clinical role

- Strongly disagree
- Disagree
- Undecided
- Agree
- Strongly agree

Appropriateness of Therapeutic Virtual Reality (Non-Clinical Role)

The following statements are about how appropriate you think the implementation of therapeutic virtual reality in private mental health services is.

1. Therapeutic virtual reality seems fitting to use in private mental health services

- Strongly disagree
- Disagree
- Undecided
- Agree
- Strongly agree

2. Therapeutic virtual reality therapy seems suitable to use in private mental health services

- Strongly disagree
- Disagree
- Undecided
- Agree

3. Therapeutic virtual reality seems applicable to use in private mental health services

- Strongly disagree
- Disagree
- Undecided
- Agree
- Strongly agree

4. Therapeutic virtual reality therapy seems like a good match with private mental health services

- Strongly disagree
- Disagree
- Undecided
- Agree
- Strongly agree

Feasibility of Therapeutic Virtual Reality (Clinical Role)

The following statements are about feasible the implementation of therapeutic virtual reality would be in your clinical role.

1. Therapeutic virtual reality seems like it would be implementable in my clinical role

- Strongly disagree
- Disagree
- Undecided
- Agree
- Strongly agree

2. It seems possible to incorporate therapeutic virtual reality into my clinical role

- Strongly disagree
- Disagree
- Undecided
- Agree
- Strongly agree

3. Therapeutic virtual reality seems like it would be easy to use in my clinical role

- Strongly disagree
- Disagree
- Undecided
- Agree
- Strongly agree

4. Introducing therapeutic virtual reality into in my clinical role seems doable

- Strongly disagree
- Disagree
- Undecided
- Agree
- Strongly agree

Feasibility of Therapeutic Virtual Reality (Non-Clinical Role)

The following statements are about feasible the implementation of therapeutic virtual reality would be in private mental health services.

1. Therapeutic virtual reality seems implementable to private mental health services

- Strongly disagree
- Disagree
- Undecided
- Agree
- Strongly agree

2. It seems possible to incorporate therapeutic virtual reality into private mental health services

- Strongly disagree
- Disagree
- Undecided
- Agree
- Strongly agree

3. Introducing therapeutic virtual reality into private mental health services seems doable

- Strongly disagree
- Disagree
- Undecided
- Agree
- Strongly agree

4. Therapeutic virtual reality seems easy to use in private mental health services

- Strongly disagree
- Disagree
- Undecided
- Agree
- Strongly agree

Please share your thoughts about using therapeutic virtual reality

1. What do you think about the introduction of therapeutic virtual reality in mental health care? What, if any, are your concerns?

2. The usefulness of therapeutic virtual reality in my clinical role would be limited by:

3. Therapeutic virtual reality would be useful in my clinical role because:

4. The use of therapeutic virtual reality in this mental health service would be limited by:

5. Therapeutic virtual reality would be useful in this mental health service because:

Survey complete. Thank you very much for sharing your thoughts and opinions.

If you would like to enter the draw for a $200 Coles Myer voucher please enter your email address here: _________________________
Your email address will not be linked to any of your survey responses.

For further reading on therapeutic virtual reality please see here for popular media summary:

https://www.theguardian.com/science/blog/2017/mar/22/why-virtual-reality-could-be-a-mental-health-gamechanger

And here for a recent academic review paper:

Freeman et al, (2017) Virtual reality in the assessment, understanding, and treatment of mental health disorders. Psychological Medicine, **47**, 2393 – 2400.

https://www.cambridge.org/core/journals/psychological-medicine/article/virtual-reality-in-the-assessment-understanding-and-treatment-of-mental-health-disorders/A786FC699B11F6A4BB02B6F99DC20237/share/5a605c8352ba7d95dddf3871e4de456f0d633372
